# Intrinsic frequency response patterns in mechano-sensory neurons of the leech

**DOI:** 10.1242/bio.023960

**Published:** 2017-05-25

**Authors:** Linda Fischer, Frank Scherbarth, Boris Chagnaud, Felix Felmy

**Affiliations:** 1 Institute of Zoology, University of Veterinary Medicine Hannover, Buenteweg 17, Hannover 30559, Germany; 2 Department Biology II, Ludwig-Maximilians-University Munich, Großhadener Straße 2, Planegg/Martinsried 82152, Germany

**Keywords:** Input-output function, Sensory filter, Neuronal excitability, Sensory integration, Medicinal leech

## Abstract

Animals employ mechano-sensory systems to detect and explore their environment. Mechano-sensation encompasses stimuli such as constant pressure, surface movement or vibrations at various intensities that need to be segregated in the central nervous system. Besides different receptor structures, sensory filtering via intrinsic response properties could provide a convenient way to solve this problem. In leech, three major mechano-sensory cell types can be distinguished, according to their stimulus sensitivity, as nociceptive, pressure and touch cells. Using intracellular recordings, we show that the different mechano-sensory neuron classes in *Hirudo medicinalis* differentially respond supra-threshold to distinct frequencies of sinusoidal current injections between 0.2 and 20 Hz. Nociceptive cells responded with a low-pass filter characteristic, pressure cells as high-pass filters and touch cells as an intermediate band-pass filter. Each class of mechano-sensory neurons is thus intrinsically tuned to a specific frequency range of voltage oscillation that could help segregate mechano-sensory information centrally.

## Abstract (precis)

**Summary:** Mechano-sensitive neurons of leech are intrinsically tuned to generate somatic input-output functions with distinct filter properties.

## INTRODUCTION

Mechano-sensory systems inform animals about the physical nature of their surroundings. The presence of a variety of mechano-sensory systems across and within vertebrate and invertebrate species reflects the importance to discriminate different forms and intensities of mechanical stimuli. In the leech, mechano-sensory neurons are classified according to their sensory threshold into nociceptive (N) cells requiring strong stimulation, pressure (P) cells requiring intermediate stimulation, and touch (T) cells requiring weak stimulation (Nicholls and Baylor, 1968; Pinato and Torre, 2000; Rodriguez et al., 2004). However, there is substantial response overlap between strong and weak stimulation intensities for static mechanical activation of these sensory neurons (Nicholls and Baylor, 1968; Pinato and Torre, 2000). T and P cells, for instance, share a range of common stimulation intensities from ∼10 to 200 mN (Kretzberg et al., 2016; Pirschel and Kretzberg, 2016). Comparing response thresholds between all three sensory cells (P, N and T cells), reveals an overlap from∼50 to 200 mN (Kretzberg et al., 2016) Thus, the different mechanical stimulation intensities potentially generate overlapping activity within the leech central neuronal network and, especially for N and P cells, it is unclear how activity is segregated. For the detection of nonstatic mechanical stimulations, T cells appear as a main source, as they were previously classified as velocity detectors (Carlton and Mcvean, 1995).

One possible way to segregate sensory information is based on the ability of neurons to encode different stimulation features through different coding strategies, such as temporal and rate coding. Thereby, the temporal response features carry more sensory information regarding the stimulation location than the spike counts, which rather encode intensities (Pirschel and Kretzberg, 2016; Thomson and Kristan, 2006). Thus, based on such multiplexing (Pirschel and Kretzberg, 2016), stimulation features and intensities might be segregated between different neuronal cell types. The tuning of intrinsic response properties might also be crucial for the sensitivity of sensory systems to specific stimulations. The intrinsic properties originate from the interaction of passive and active membrane properties, thereby defining the characteristic responses of a given neuron (Franzen et al., 2015). Characteristic response patterns, such as accommodation patterns (Schlue, 1976a) or temporal precision (Ammer et al., 2012; Franzen et al., 2015), might provide cues for processing different sensory inputs centrally. An arrangement of different intrinsic response properties within a sensory system is thus potentially able to generate a filter bank that could support the segregation of sensory inputs, or might support segregation of information processing. One crucial intrinsic property of neurons is their ability to follow specific stimulation frequencies. To assess the stimulation frequency that a neuron responds best to, the injection of sinusoidal currents of different frequencies is well suited.

Here, we tested the hypothesis that N, P and T cells have different intrinsic frequency response patterns. Our somatic recordings demonstrate that each class of sensory neurons displays different filter characteristics: low-, band- and high-pass filters. From this finding, we speculate that such an intrinsic filter bank of sensory neurons could support the central segregation of mechano-sensory information.

## RESULTS AND DISCUSSION

The intrinsic response characteristics of the three main types of mechano-sensory neuron to sinusoidal current injections at frequencies between 0.2 and 20 Hz were investigated in *Hirudo medicinalis*. Sinusoidal current injections can be used to approximate the input-output characteristics of neurons. This approximation does not necessarily reflect the peripheral sensory input; instead it highlights the central processing capabilities.

**Fig. 1.**
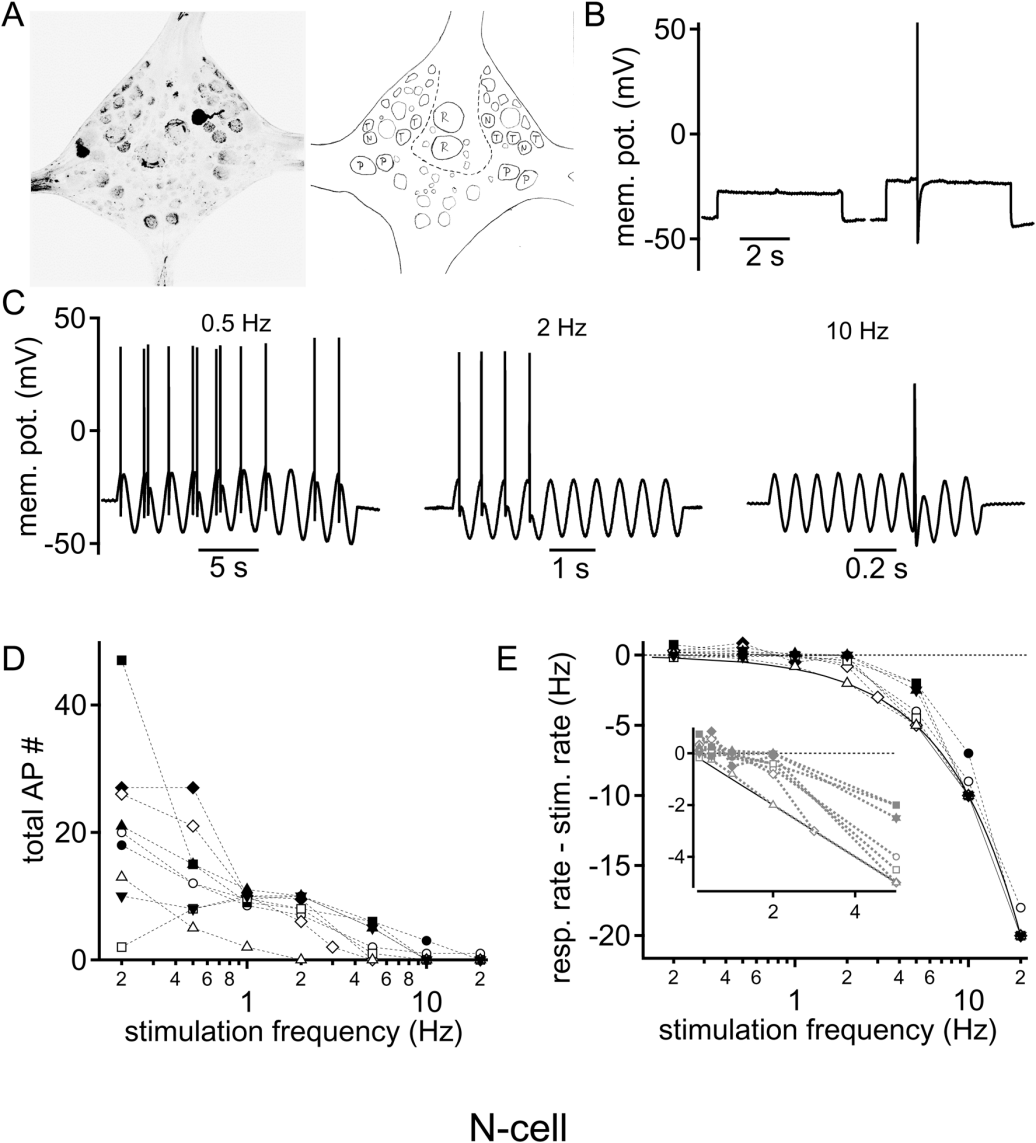
**Intrinsic frequency response profile of N cells to sinusoidal current injections.** (A) Fluorescent labeling of a recorded N cell was used to identify the cell location post-hoc by comparison to a schematic drawing of the known location of leech sensory neurons. (B) Square pulse current injection just below (left) and above (right) the action potential threshold. Action potential waveform shows the N cell characteristics long after hyperpolarization. (C) Voltage responses to different sinusoidal stimulation frequencies: left, 0.5 Hz; middle, 2 Hz; right, 10 Hz. (D) Number of supra-threshold responses summed over the 10 sinusoidal cycles as a function of stimulation frequency. Each symbol represents the response of a single cell (*n*=9). (E) Action potential firing rate from which the stimulation rate was subtracted is plotted as a logarithmic function of the stimulation frequency. The dotted zero line indicates the same firing rate as the number of stimulation cycles, hence faithful firing. The solid line represents the zero action potential line, where no supra-threshold response was elicited at any time during stimulation. Symbols are as in D. Inset, magnified low stimulation frequencies in a linear graph.

N cells show a high sensory threshold, as relatively strong forces must be applied to the skin to elicit their supra-threshold response (Lewis and Kristan, 1998; Nicholls and Baylor, 1968; Pinato and Torre, 2000). Here, N cells were identified by their characteristic location (Nicholls and Baylor, 1968; Yau, 1976) (Fig. 1A) and action potential after-hyperpolarization (Fig. 1B). Recorded N cells had an average resting membrane potential of −41.3±2.7 mV (*n*=9). Sinusoidal current injections of 10 cycles ranging from 0.2 to 20 Hz were applied to the somatic region. The current amplitude was adjusted to regularly elicit action potentials to a current sinewave delivered at 1 Hz (i.e. slightly over threshold). N cells responded in a frequency-dependent fashion. The number of evoked action potential decreased with increasing stimulation frequency (Fig. 1C). The average of the maximally fired number of action potentials (20.4±4.2 Hz; *n*=9) was elicited at 0.2 Hz. In only one of nine cells, the maximal action potential number was elicited at 1 Hz, not at 0.2 Hz. At 20 Hz, only one of these cells was still able to generate an action potential (Fig. 1D). To compare the firing behavior across frequencies in more detail, we first compensated for the duration of stimulation by calculating the action potential frequency. Second, we subtracted this response rate from the stimulation frequency. This analysis generates positive values when multiple action potentials occur during a single stimulation cycle (Fig. 1E). When no action potential is elicited, a value equal to the stimulation frequency is obtained (Fig. 1E). This analysis showed that N-cells only responded with multiple action potentials to sine-stimulations <1 Hz, with one action potential at 1 Hz, and stopped responding at an average rate of 6.2±2.3 Hz. Taken together, these results indicated that the N cells responded with low-pass filter properties to sinusoidal stimulations.

**Fig. 2.**
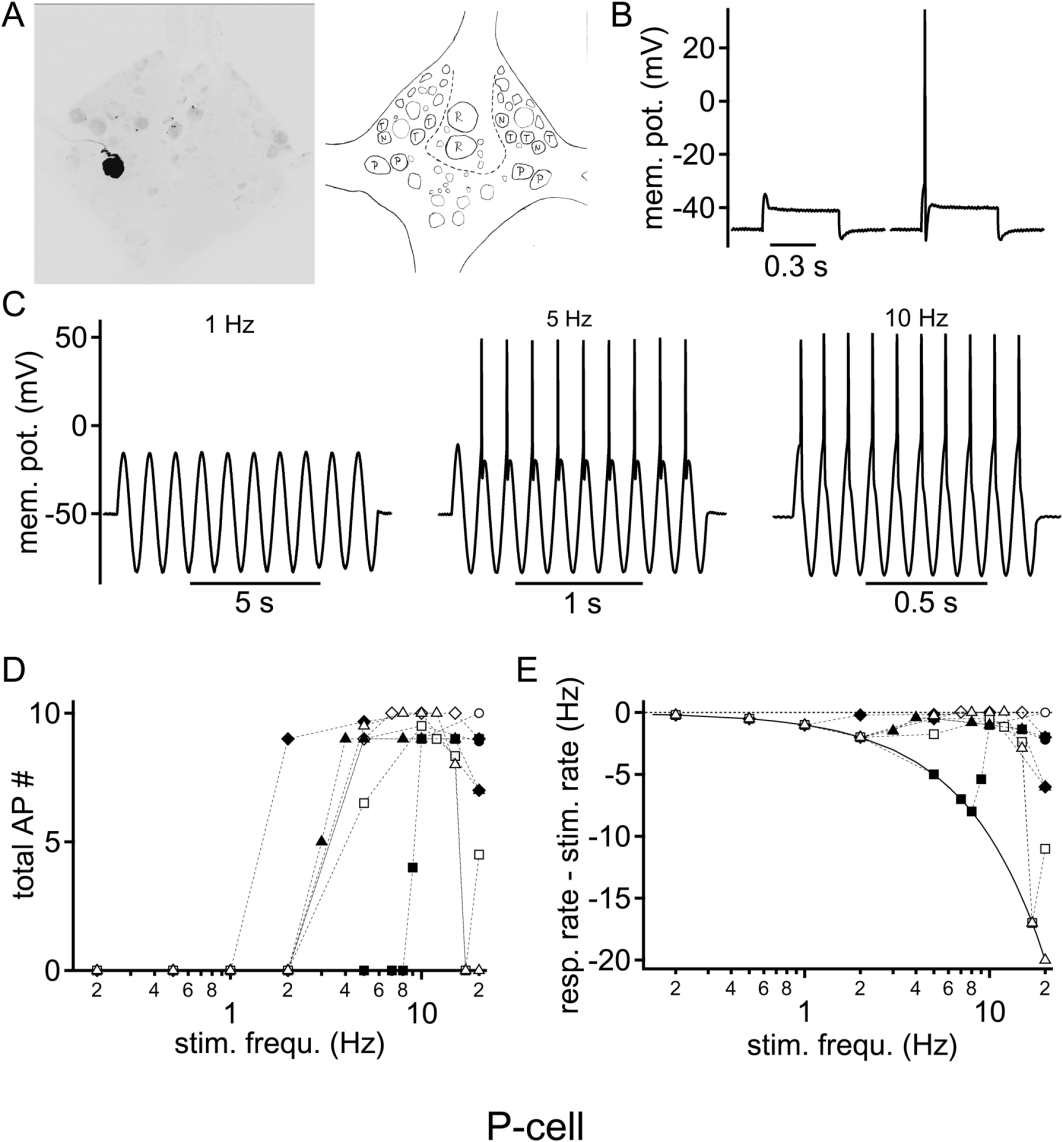
**Intrinsic frequency response profile of P cells to sinusoidal current injections.** (A) Fluorescent labeling of a recorded P cell was used to identify the cell location post-hoc by comparison to a schematic drawing of the known location of leech sensory neurons. (B) Square pulse current injection just below (left) and above (right) the action potential threshold. The rapid onset action potential is characteristic of P cells. (C) Voltage responses to different sinusoidal stimulation frequencies: left, 1 Hz; middle, 5 Hz; right, 10 Hz. (D) Number of supra-threshold responses summed over the 10 sinusoidal cycles as a function of stimulation frequency. Each symbol represents the response of a single cell (*n*=8). (E) Action potential firing rate from which the stimulation rate was subtracted is plotted as a logarithmic function of the stimulation frequency. The dotted zero line indicates the same firing rate as the number of stimulation cycles, hence faithful firing. The solid line represents the zero action potential line, where no supra-threshold response was elicited at any time during stimulation. Symbols are as in D.

**Fig. 3.**
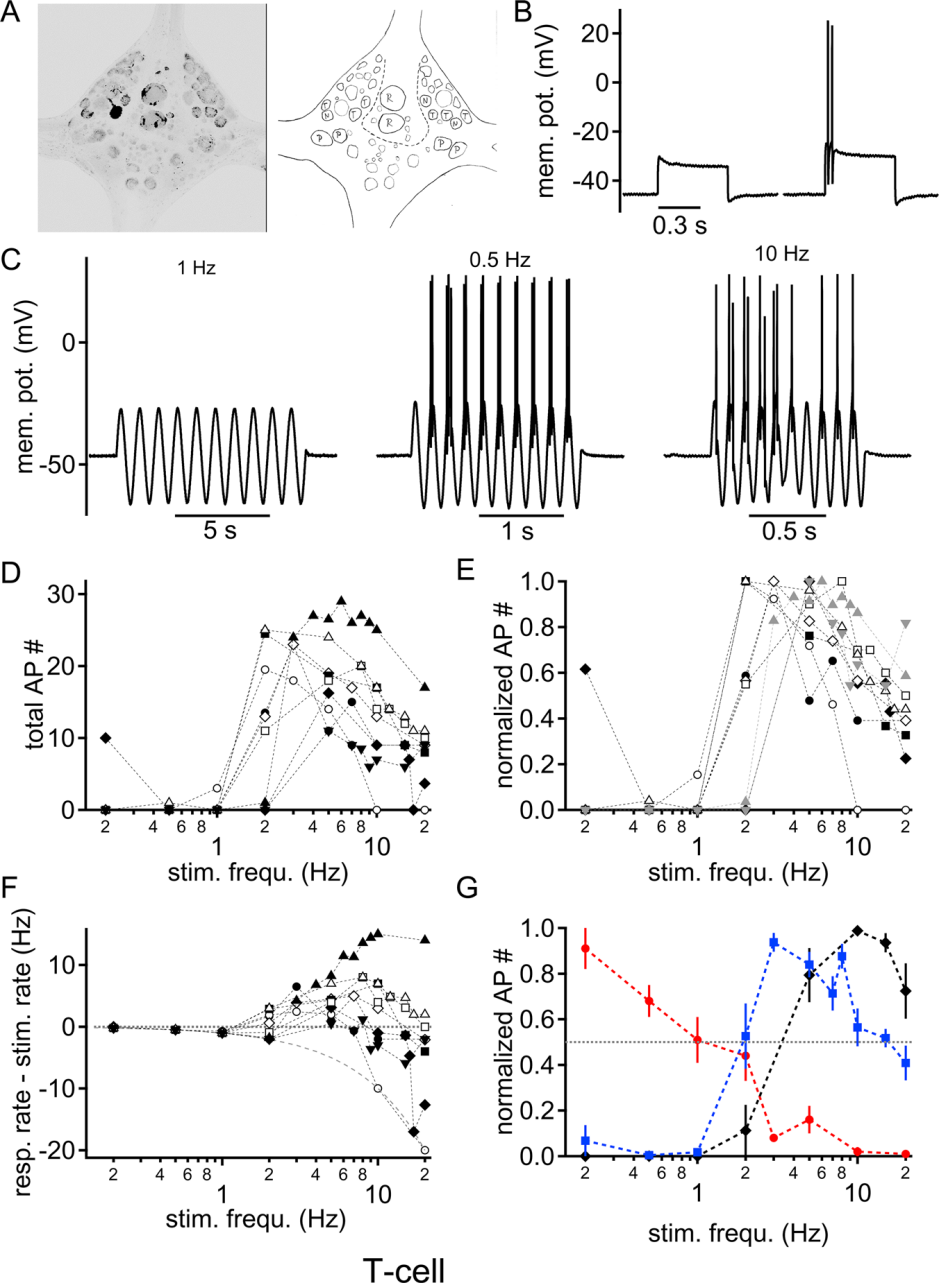
**Intrinsic frequency response profile of T cells to sinusoidal current injections.** (A) Fluorescent labeling of a recorded T cell was used to identify the cell location post-hoc by comparison to a schematic drawing of the known location of leech sensory neurons. (B) Square pulse current injection just below (left) and above (right) the action potential threshold. The initial burst of action potential is characteristic of T cells. (C) Voltage responses to different sinusoidal stimulation frequencies: left, 1 Hz; middle, 0.5 Hz; right, 10 Hz. (D) Number of supra-threshold responses summed over the 10 sinusoidal cycles as a function of stimulation frequency. Each symbol represents the response of a single cell (*n*=9). (E) Normalized number of action potentials as a function of stimulation frequency. The dotted horizontal line indicates half maximal firing. Symbols are as in D. (F) Action potential firing rate from which the stimulation rate was subtracted is plotted as a logarithmic function of the stimulation frequency. The dotted zero line indicates the same firing rate as the number of stimulation cycles, hence faithful firing. The solid line represents the zero action potential line, where no supra-threshold response was elicited at any time during stimulation. Symbols are as in D. (G) Normalized action potential firing in response to different sinusoidal stimulation frequencies of injected current for N (red), P (black) and T (blue) cells. Only stimulation frequencies where at least four cells were recorded are presented (mean±s.d.).

P cells are classically characterized by their intermediate to high sensory threshold to skin stimulation (Lewis and Kristan, 1998; Nicholls and Baylor, 1968; Pinato and Torre, 2000), and therefore their sensory threshold overlaps substantially with N cells. Here, P cells were identified by their characteristic location (Nicholls and Baylor, 1968; Yau, 1976) and their onset response to strong stimulation with a square pulse current (Fig. 2A,B). The average P cell membrane resting potential of −46.6±1.6 mV (*n*=8) resembled that reported previously (Schlue and Deitmer, 1984). The strength of sinusoidal stimulation intensity was adjusted to reliably elicit action potentials at a frequency of 5 Hz (Fig. 2C), as this appeared to be the lowest frequency at which low amplitudes of injected current were sufficient to drive P cells efficiently. At this intensity, low stimulation frequency failed to generate supra-threshold responses. The lowest stimulation frequency that elicited action potentials in P cells was 4.6±0.7 Hz, on average (*n*=8) (Fig. 2D,E). P cells, however, followed increasing sinusoidal stimulation frequency with reliable action potential firing largely up to ∼15 Hz. The maximal response of P cells was found at an average stimulation frequency of 9.8±1.6 Hz. However, in only one of eight recorded P cells, did the response rate decrease below 50% at higher stimulation frequencies (Fig. 2D,E). Overall, the P cell response dropped by only 25% (Fig. 3G) at a stimulation frequency of 20 Hz. Taken together, these results indicated that P cell membrane properties can be regarded as acting as an intrinsic high-pass filter.

T cells have a lower sensory stimulation threshold compared to N and P cells (Lewis and Kristan, 1998; Nicholls and Baylor, 1968; Pinato and Torre, 2000). Furthermore, T cells are characterized by location (Nicholls and Baylor, 1968; Yau, 1976), and by a bursting onset response to square pulse current injections (Kretzberg et al., 2007; Schlue, 1976a,b). We used the location and onset bursting to identify T cells (Fig. 3A,B). The average T cell resting potential was −42.6±1.2 mV (*n*=9). For the applied sine wave current injections, the intensity was adjusted to elicit reliable firing between 2 and 5 Hz (Fig. 3C). Again, these stimulation frequencies appeared to be the lowest to evoke action potentials with sinusoidal stimulations frequencies efficiently with low current amplitudes. Using this approach, only one of nine T cells responded to stimulation rates below 1 Hz. The lowest frequency that generated supra-threshold excitation was 1.85±0.46 Hz, on average. The maximal number of action potentials was elicited at an average stimulation frequency of 4.0±0.7 Hz (Fig. 3D,E). Here, the frequency of action potentials exceeded the stimulation frequency and was 6.2±1.4 Hz (Fig. 3F). At high frequencies, the number of action potentials elicited decreased for all cells tested (Fig. 3D); in only two of the nine T-cells the response remained above 50% and in six cells the response dropped below one action potential per stimulation cycle (Fig. 3E). The average stimulation frequency that still elicited 50% of maximal firing rate was 12.6±2.6 Hz, on average. It is worthy to note that in six of these cells the action potential response steadily declined. T cells thus responded with elevated firing rates to sinusoidal modulated voltage deflections between 4 and 12 Hz, and their overall ability to rate code might therefore be intrinsically tuned to behave as a band pass filter.

We have here identified different, frequency-dependent supra-threshold response patterns based on firing rates for mechano-sensory neurons in *Hirudo medicinalis*. N cells responded preferentially to low, P cells to high, and T cells to intermediate stimulation frequencies, illustrating a differential intrinsic tuning to sinusoidal current stimulations (Fig. 3G). These intrinsic properties thus appear to generate specific low-, band- and high-pass filters for supra-threshold firing rates, respectively.

**Fig. 4.**
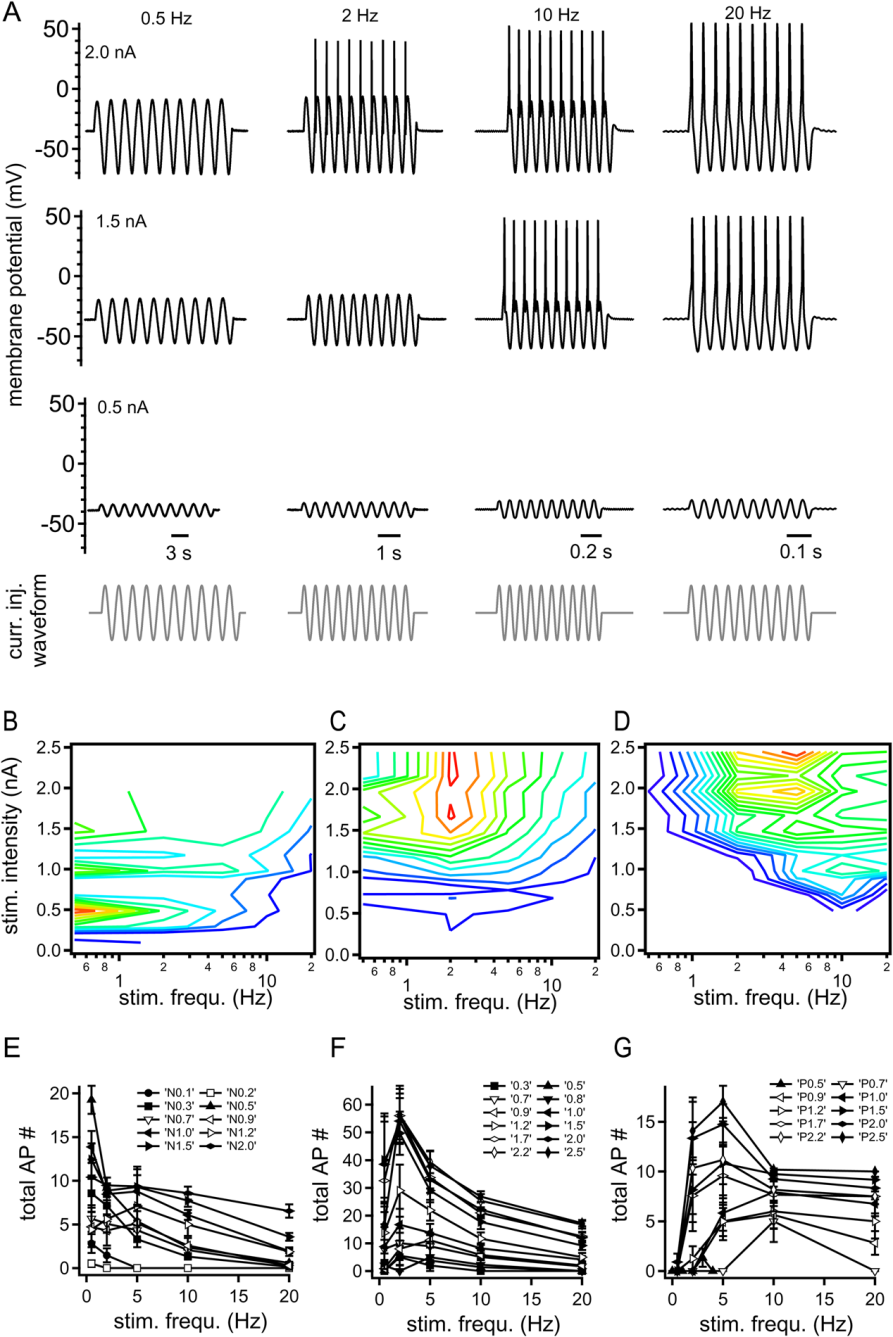
**Supra-threshold response profiles are different in N, T and P cells, and depend on stimulation intensity and frequency.** (A) Sub- and supra-threshold membrane potential responses (black) of a P cell to current injections (grey) of 0.5 Hz (left), 2 Hz (left center), 10 Hz (right center) and 20 Hz (right) at stimulation intensities of 0.2 nA (top), 0.15 nA (middle) and 0.05 nA (bottom). (B-D) Number of action potentials plotted as a function of stimulation intensity and frequency for (B) N, (C) T and (D) P cells. Line color spectrum from dark blue to red represents the number of action potentials from low to high numbers. (E-G) Number of action potentials elicited in response to current injections of different stimulation intensities: (E) N cell: 0.1, 0.2, 0.3, 0.5, 0.7, 0.9, 1.0, 1.2, 1.5 and 2.0 nA (*n*=4-21); (F) T cell: 0.3, 0.5, 0.7, 0.8, 0.9, 1.0, 1.2, 1.5, 1.7, 2.0, 2.2 and 2.5 nA (*n*=4-16); and (G) P cell: 0.5, 0.7, 0.9, 1.0, 1.2, 1.5, 1.7, 2.0, 2.2 and 2.5 nA as a function of the stimulation frequency (0.5, 2, 5, 10 and 20 Hz). Error bars represent s.e.m.

To verify that the intrinsic membrane properties of mechano-sensory neurons generate different filters, we once more recorded from all three mechano-sensory neurons; however this time varying the stimulation intensity over a reduced range of stimulation frequencies (Fig. 4). For the stimulation frequencies of 0.5, 2, 5, 10 and 20 Hz, the intensity ranged from 0.1 to 2.5 nA. Fig. 4A depicts a subset of the P cell responses when challenged with this stimulus matrix of sinusoidal current injections. The stimulation matrix was used to determine whether the different filter responses of the N, T and P cells are independent of stimulation intensity, and to see how the response profiles segregate. For this reason, the action potential number was extracted from each sinusoidal current injection and given as color-coded intensity in Fig. 4B-D. With increasing stimulation intensity, N cells responded with increasing number of action potentials especially at low stimulation frequencies (Fig. 4E). At high stimulation intensities, the voltage excursion became so large that N cells could not fire action potentials or the action potentials were masked. At high stimulation intensities, therefore, supra-threshold N cell responses are missing. T cells predominantly increased action potential firing with increasing stimulation intensities at intermediate stimulation frequencies with a maximum firing locked to 2 Hz (Fig. 4C). T cell firing remained lower at higher stimulation frequencies. For P cells, the increase in stimulation intensity led to increased firing starting at frequencies of 10 Hz, which became maximal at 5 Hz for strong stimulation intensities (Fig. 4D). Importantly, and in contrast to T and N cells, P cells were never excited at low frequencies. Presenting the number of action potentials as discrete functions of stimulation frequencies (Fig. 4E-G) illustrates the same finding. Taken together, these results indicated that N, T and P cells generate intrinsically a low-, band- and high-pass filter, respectively.

Mechano-sensory cells in leech are directly linked to mechanical transduction at the skin surface, as quenching synaptic transmission does not suppress mechano-sensory signaling in these neurons (Burgin and Szczupak, 2003; Nicholls and Baylor, 1968). Thus, mechano-sensory transduction is supposed to drive N, T and P cells directly. Assuming that voltage signaling at the periphery and the soma differs only based on compartment size, but not on active membrane properties, our observed excitability profiles allow insights into the sensory input-output transformation. The low-, band- and high-pass filter characteristics would therefore contribute to the distribution of mechano-sensory information into a central neuronal filter bank in leech. Such central filtering might be suited to segregate strong sensory stimulations that might evoke activity in each mechano-sensory cell type, especially the N and P cells.

We describe a difference in the intrinsic frequency-dependent excitability profile of mechano-sensory cells. In general, ionic conductance (Ratte et al., 2013) and cell morphologies (Mainen and Sejnowski, 1996) influence the characteristic intrinsic response patterns of neurons (Franzen et al., 2015). Besides a different morphology, the influence of which remains here enigmatic, mechano-sensory neurons in leech show differences in the strength of expressed ionic conductances. P cells have the strongest hyperpolarization activated conductance (Gerard et al., 2012), and their delayed potassium rectifier gates faster compared to N cells (Stewart et al., 1989). Both findings support the faster voltage signaling in P cells compared to N cells that we describe. N cells show the strongest and slowest action potential after-hyperpolarization of mechano-sensory neurons (Schlue, 1976a), which possibly quenches firing at high stimulation rates, thus turning these neurons into a low-pass filter. Conversely, the P cell high-pass characteristic might be enabled by a fast-delayed rectifier paired with a hyperpolarization-activated current and a small and shorter after-hyperpolarization. For P cells, additionally an inactivation of the somatic action potential generator by slow evolving depolarizations has to be postulated to suppress activation at low input frequencies. This difference in action potential generation might be supported by the distinct sodium expression profiles in the mechano-sensory neurons in leech (Blackshaw et al., 2003). However, the detailed differences in ionic conductances and their local expression profile generating the differences in the described intrinsic response profiles between N, T and P cells remain so far unclear.

In conclusion, our study shows that the intrinsic response patterns of somata of mechano-sensory neurons in leech represent an intrinsic filter bank in respect to the ideal input frequency. These different characteristic response patterns might be suited to support segregation of overlapping sensory information centrally, possibly in conjunction with the different stimulus sensitivities and central connectivities. How these intrinsic features interact with actual sensory stimulation of different frequencies at the periphery remains to be shown.

## MATERIALS AND METHODS

Leeches (*Hirudae medicinalis*) were ordered from HiroCult (Willhelmshaven, Germany) and kept in fresh water at 7-18°C. No animal license is required by the local authorities for work on leeches. To prepare ganglions, the leeches were taken out of the fresh water, opened dorsally under binocular vision in fridge-cooled (∼6°) Ringer solution (115 mM NaCl, 4 mM KCl, 1.8 mM CaCl_2_, 1.5 mM MgCl_2_, 10 mM glucose, 4.6 mM Tris maleate and 5.4 mM Tris base adjusted to pH 7.4 using NaOH). Extracted ganglions were fixed ventral side up in Sylgard plated dishes using small minutien pins (0.1 mm diameter).

Intracellular voltage signals were recorded in current clamp mode using an IE 251A amplifier (Warner Instruments, Hamden, USA) connected to a PowerLab 26T (ADInstruments, Oxford, UK) controlled by LabChart8 (ADInstruments) on a standard personal computer. An acquisition rate of 20 kHz was used to sample the voltage responses. Current stimulation was controlled by LabChart8 allowing sinusoidal stimulation between 0.2 and a maximal rate of 20 Hz. This stimulation range covers at least the frequency range during undulatory swimming (Gray et al., 1938; Kristan et al., 1974; Stent et al., 1978), i.e. reflecting a large part of the likely experienced mechanical stimulation frequencies of the animal. Mechanical activation at high frequencies (≤800 Hz), as in primates (Harvey et al., 2013), is likely less important in leech since the behaviorally relevant frequency range, e.g. of water waves, is lower (Harley et al., 2011). Intracellular electrodes with 30-50 MΩ resistances were pulled from borosilicate glass using a P-97 puller (Sutter Instrument, Novato, USA). In Figs 1 to 3, reliable action potential responses were stimulated with low current amplitudes. Therefore, the stimulation frequency was chosen where a low current amplitude reliably elicited one action potential per cycle. This stimulation frequency/intensity matching differed between the three classes of cells investigated. In Fig. 4, a largely common range of stimulation intensities was applied to each cell at standard frequencies of 0.5, 2, 5, 10 and 20 Hz. Here, only cells were taken into account where at least four different stimulation intensities for the standard stimulation frequencies were recorded. Moreover, additional stimulation frequencies were only then taken into account when recordings from at least four different cells were obtained. A 2 M KCl solution was used for intracellular recordings to which 0.5 mM Alexa 488 or Alexa 594 (Thermo Fisher Scientific) was added to visualize cell morphology and location post hoc. Presented images are image stacks obtained with a confocal microscope (Leica TCS SP5, Leica Microsystems, Wetzlar, Germany). Data were analyzed using LabChart8, Igor Pro 6 (WaveMetrics; https://www.wavemetrics.com/products/igorpro/newfeatures/previous/upgradereasons63.htm) and Microsoft Excel. Data are presented as mean±standard error of the mean.
